# Relationship of Parental Support on Healthy Habits, School Motivations and Academic Performance in Adolescents

**DOI:** 10.3390/ijerph17030882

**Published:** 2020-01-31

**Authors:** José Enrique Moral-García, José David Urchaga-Litago, Antonio Jesús Ramos-Morcillo, Rubén Maneiro

**Affiliations:** 1 Physical Activity and Sports Sciences, Faculty of Education, Pontifical University of Salamanca, Calle Henry Collet, 52-70, 37007 Salamanca, Spain; jemoralga@upsa.es; 2 Faculty of Communication, Pontifical University of Salamanca, Calle Henry Collet, 90-98, 37007 Salamanca, Spain; 3 Department of Nursing, Faculty of Nursing, University of Murcia, 30100 Espinardo, Spain; ajramos@um.es

**Keywords:** parental influence, physical activity, school performance, alcohol consumption, adolescents, age differences, gender differences

## Abstract

The objective of the study was to analyze how parental support relates to the physical activity practice, satisfaction with sports, level of physical activity, academic performance and alcohol consumption. Descriptive cross-sectional study, with 1100 adolescents (12–16 years old), where the factors related to parental support, gender and age acted as independent variables, and satisfaction with sport, level of physical activity (PA), academic performance and alcohol consumption acted as dependent variables. A multivariate statistical analysis was conducted. Adolescents with little parental support show (*p* < 0.001) more boredom, less fun, worse academic performance and higher alcohol consumption. Gender shows differences (*p* < 0.001) experiencing girls more boredom, less fun, less PA practice and higher academic performance than boys. Age establishes (*p* < 0.01) that older adolescents (15–16 years old) experience more boredom, less fun, less PA practice, lower academic performance and higher alcohol consumption than young boys and girls (12–14 years old). Parental support towards PA practice improves healthy habits, benefits academic performance and school satisfaction with physical and sports activity.

## 1. Introduction

The parental influence on the behaviors of their teenage children can be related to the theory of social learning [1] projecting this influence on the physical activity (PA) practice by the implicit socialization component existing within it [2]. In addition, the influence exerted by paternal stimulation on the future PA practice by adolescents, is also reinforced by the importance that schoolchildren grant to physical education (PE) [3] and by the satisfaction or boredom experienced by these teenagers in the PE class [4]. In fact, school boredom in PE may be related to lower levels of future PA practice [5,6].

In order to explain the reasons why an individual decides to perform PA, it is necessary to take into account the motivation. This motivation allows us to understand the existing internal or external factors, which will favor the appearance or maintenance of a behavior or action [7]. This can be explained from the social cognitive theory [8] and the theory of self-determination [9], classifying motivation from three perspectives: intrinsic, extrinsic or not motivated [10]. 

Parents have a very important responsibility in terms of promoting and strengthening their children’s PA practice [11,12]. Raise awareness among parents regarding the fact that all support they provide to their children is a generator of adherence to PA and maintenance of sports participation in the future, is crucial [13]. Often, it is unknown that this influence can be expressed in different ways [1]. The most evident way is due to the example of physically active parents [14], but some others include the support and encourage of the children to practice PA [14,15], without forgetting their role as financial support [15]. Therefore, it is important that parents to be interested in the use of their children’s free time, so that PA practice acquires a greater presence in their future [13].

Currently, parental support acquires greater relevance since adolescents invest a lot of time every day using screen devices [16], a circumstance that is aggravated when both parents act as a negative example, also making excessive use of these devices and having a sedentary leisure [17]. Although parental support is essential in both genders, it seems that maternal modeling of healthy habits has a greater influence on children compared to paternal modeling [17,18].

Adolescence is a decisive age period for the generation and consolidation of a healthy lifestyle, which inevitably needs a PA practice on a regular basis [19], being aware that positive habits consolidated at an early age have an impact on the adulthood [20]. Some of the benefits of PA practice include the prevention or improvement in certain diseases such as cardiovascular diseases, the improvement of physical condition [21], benefits at the psychosocial level such as the promotion of self-esteem, reduction of depression and stress [22], cognitive development [23,24], socialization and academic performance [25,26]. However, at present, PA practice does not comply with the recommendations established for adolescents [27], due to, among other reasons, the increasing use of new technologies and display devices [28,29]. Therefore, there is a higher risk that current adolescents become sedentary or less active adults [30].

Alcohol consumption is a major health problem currently, especially at adolescence [31,32,33]. There is no strong scientific evidence to ensure that the parents’ alcohol supply to their teenage children is an effective strategy that reduces the prevalence of consumption at later ages. On the contrary, this practice of parental support for alcohol consumption poses a risk to the health of their children (to their children’s health) [34]. Excessive alcohol consumption in parents may be a risk factor for excessive consumption in children [35]. It seems that mothers have more influence on their children’s alcohol consumption compared to fathers [36]. In a recent study, it has been found that adolescents who practice more physical activity, enjoy physical-sporting practice more, consume less alcohol and have better healthy habits [37].

Despite the evidences proving the positive influence of parental support on the adherence to PA practice [11,12], not all studies appreciate this connection [38]. Therefore, and being aware that there are not enough previous studies relating parental support towards PA practice with the school sports satisfaction, with healthy habits and academic performance, this paper aims to study how parental support influences on PA practice, on the satisfaction with school sports, the level of PA, academic performance and alcohol consumption.

## 2. Materials and Methods 

### 2.1. Design and Participants

A descriptive and cross-sectional study was designed, with a total of 1100 Spanish adolescents (55.1% girls), aged 12–16 years old (14.36 ± 1.61 years). The sample was distributed in five Compulsory Secondary Education (High School) centers of two Autonomous Communities of Spain and the data collection period was between February and May of 2019. We worked with an error <0.03 and with a confidence interval of 95%. This research respected ethical considerations established in the Declaration of Helsinki in its 2013. The rest of the ethical considerations and the inclusion and exclusion criteria, for the selection of the sample, were explained in detail in the procedure section.

### 2.2. Instruments

Socio-demographic questionnaire. It collected information on gender (male and female) and age (12, 13, 14, 15 and 16 years old).

Parental Support for Physical Activity Questionnaire (APAF, for its Spanish initials). The scale parental influence on physical activity [39], resulting from previous research, was used [40,41]. This questionnaire was made up of 14 items distributed in four subscales: general parenting support or APAF1 (1, 2, 3, 4, 5 and 6), active parents or APAF2 (7, 8, 9 and 10), past activity or APAF3 (11 and 12) and guiding support or APAF4 (13 and 14). It has a Likert-type scale with four possible options ranging from 1 (never) to 4 (always). This instrument in its original version presented a global α of 0.746 and a total explained variance of 67.5%.

Sports Satisfaction Questionnaire. The Sport Satisfaction Instrument (SSI) questionnaire adapted to physical education (PE) in Spain was used [42]. Made up of 8 items divided into two subscales: fun (1, 5, 6, 7 and 8) and boredom (2, 3 and 4). It has a Likert-type response scale of 5 options ranging from 1 (strongly disagree) to 5 (strongly agree). The internal consistency of the fun subscale was α = 0.92 and of the boredom subscale α = 0.79.

PA Practice Questionnaire. To analyze the level of PA practice, from moderate to vigorous, the questionnaire “MVPA” [43] was used. It consisted of two items that collect information on the days per week when at least 60 minutes of moderate to vigorous PA is practiced, both in the previous week and in a typical week. The response scale for both was the same (0 = no day, 1 = one day, 2 = two days, 3 = three days, 4 = four days, 5 = five days, 6 = six days and 7 = seven days). In this research, the average of both items was obtained [44,45].

Alcohol Consumption Questionnaire. This instrument was extracted from the Alcohol Consumption Scale Questionnaire [46], adapted to Spanish [47]. For this study, only the item related to the quantity and frequency of alcohol consumption was used, categorized into four response options: no consumption (1), low consumption (2), average consumption (3) and excessive consumption (4), as used in other researches [37].

### 2.3. Procedure

Ethical considerations. This research respected at all times the ethical considerations established in the Declaration of Helsinki in its 2013 revision as well as the Personal Data Protection Act [48]. Before the data collection setting in motion the study, school and parents or legal guardians of the participating adolescents were informed in detail about the type of study to be carried out. The anonymity and confidentiality of those involved were guaranteed at all times, through a code system. Those interviewed received no academic or monetary compensation. The principal investigator supervised the provision of the different questionnaires used. In practice, the development of the different instruments used was carried out by teachers with the concentration in physical education, who had at least ten years of teaching experience, and who had previously been trained with the different instruments and materials that were to be used.

Sample Selection. The eligible population was adolescents enrolled in educational centers included in the research with the following inclusion criteria: age between 12 and 16 years old. Among the inclusion criteria were voluntary participation with the consent of the parents or legal guardians, not to follow any food or diet restriction at the time of the study or in the previous six months and not to suffer from any disease incompatible with PA practice. The exclusion criteria were answering incorrectly or incompletely to any of the items, not presenting parental or legal guardian’s authorization, not having the authorization or approval of the school to which they belong, presenting some type of disease during the period of study incompatible with PA practice and/or which requires a restrictive diet or a specific one during the study period or in the previous six months.

### 2.4. Data Analysis

The study data were analyzed with the statistical package SPSS version 24.0 (SPSS Inc., Chicago, IL, USA). Descriptive analyses of mean values and standard deviation were conducted. Multivariate analyses (MANOVA) were performed, studying the main effects of the gender and age factors (2 × 2) and the 4 factors of the APAF questionnaire (2 × 2 × 3), with boredom, fun, PA practice, academic performance and consumption of alcohol as dependent variables. Wilks’ lambda, significance and the partial eta squared were calculated.

## 3. Results

### 3.1. Descriptive Analysis

The table below (Table 1) shows which factors were significant in relation to the different variables, the subject matter of the study. Out of the four parental support factors, the one presenting the greatest relationship with the analyzed variables was the first factor (APAF1).

The IPAF1 factor was significantly correlated to all the variables studied, showing that adolescents with little general parental support are those who get bored more (*p* < 0.001; low support M = 2.27 and high support M = 1.92), have less fun (*p* < 0.001; low support M = 3.91 and high support M = 4.56), are less physically active (*p* < 0.001; low support M = 2.56 and high support M = 3.62), have lower academic performance (*p* = 0.031; low support M = 6.80 and high support M = 7.17) and consume more alcohol (*p* < 0.001; low support M = 1.39 and high support M = 1.25).

The IPAF2 factor was significantly correlated to the variables of fun (*p* = 0.006; low support M = 4.13 and high support M = 4.31) and of alcohol (*p* < 0.001; low support M = 1.43 and high support M = 1.26), showing that high parental support of active parents was correlated to more fun and less alcohol consumption.

The IPAF3 factor, corresponding to the past parental activity, was significantly correlated to the variables of boredom (*p* = 0.003), detecting that the adolescents who were most bored were those who feel a lower support (M = 2.14) and a higher support (M = 2.18), being those who were less bored the adolescents receiving a medium support (M = 2.00); of academic performance (*p* = 0.003), having the best grades those with medium support (M = 7.12) and having the worst grades those with a higher support (M = 6.80); of alcohol consumption (*p* = 0.002), being those who drink the most, the adolescents with the lowest support (M = 1.40) and the ones who drink the least, those with a higher support (M = 1.93).

The IPAF4 factor, the guidance support, was significantly correlated to the variable of boredom (*p* = 0.001), those with high support show more boredom (M = 2.21) than those with low (M = 2.08) and medium (M = 2.00).

Gender marked differences (*p* < 0.001) in the variables of boredom, fun, PA and academic performance. Thus, girls experienced more boredom, less fun, less PA practice and greater academic performance than boys. There were no differences in alcohol consumption.

Age was significantly correlated to the variable of boredom (*p* = 0.003), fun (*p* < 0.001), PA (*p* < 0.001), academic performance (*p* < 0.001) and alcohol consumption (*p* < 0.001). The older adolescents show more boredom, less fun, less PA practice, lower academic performance and higher alcohol consumption.

### 3.2. Multivariate Analysis

The multivariate analysis shows significant effects both in the different factors and in the interactions among them, as well as in many of the dependent variables. Firstly, the main effects of the factors were analyzed and secondly, those significant and most relevant interactions (Table 2).

### 3.3. Main Effects Between Parental Support Factors with Age and Gender in Relation to Dependent Variables: Boredom, Fun, PA Practice, Academic Performance and Alcohol Consumption.

The figures shown below are a selection of the most significant interactions (*p* < 0.05) found among the different variables analyzed.

**Figure 1:**
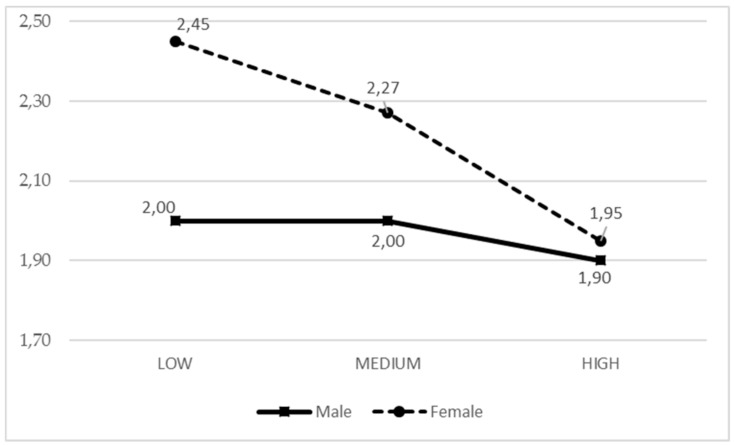
Means of the IPAF1 parental style based on the adolescent gender for boredom.

**Figure 2:**
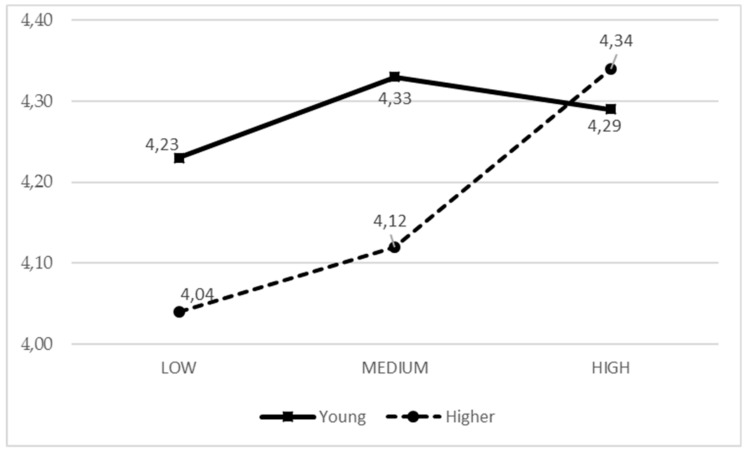
Means of the IPAF2 parental style based on the adolescent age for fun.

It was observed that when girls receive a greater general parental support, they experience less boredom, while in the case of boys, the boredom level is independent from the parental support (Figure 1). In older adolescents, more parental support from active parents equals to them experiencing more fun, while in young boys and girls, parental support was less related to fun (Figure 2).

**Figure 3:**
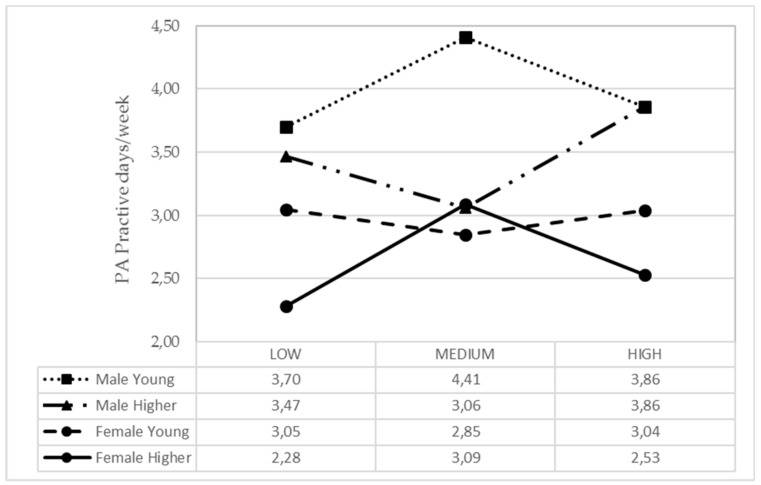
Means of the IPAF3 parental style based on the adolescent gender and age for PA practice.

**Figure 4:**
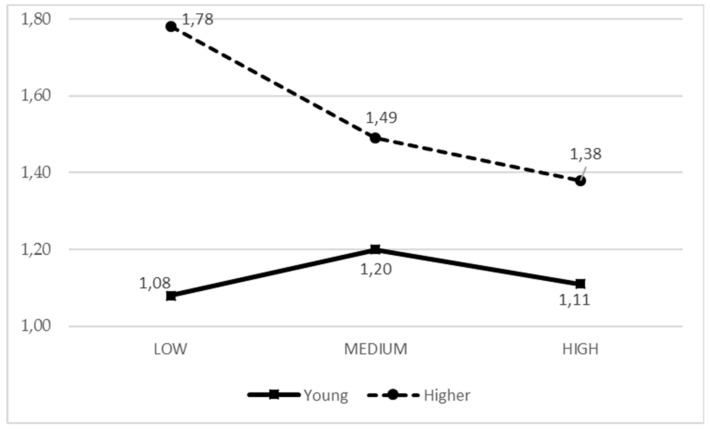
Means of the IPAF3 parental style based on the adolescent age for alcohol consumption.

In general, PA practice was high in men, especially when they were young, the moment when their parental support (from past parental activity) means are more active, while in those older the practice is independent from the parental support received. In girls, to some extent, this trend is reversed, since parental support does not influence young girls, but it does influence those older, where average parental support increases PA practice (Figure 3). In younger adolescents, alcohol consumption is lower and independent from parental support from past activity. However, with an older age, alcohol consumption increases with low parental support (Figure 4).

**Figure 5:**
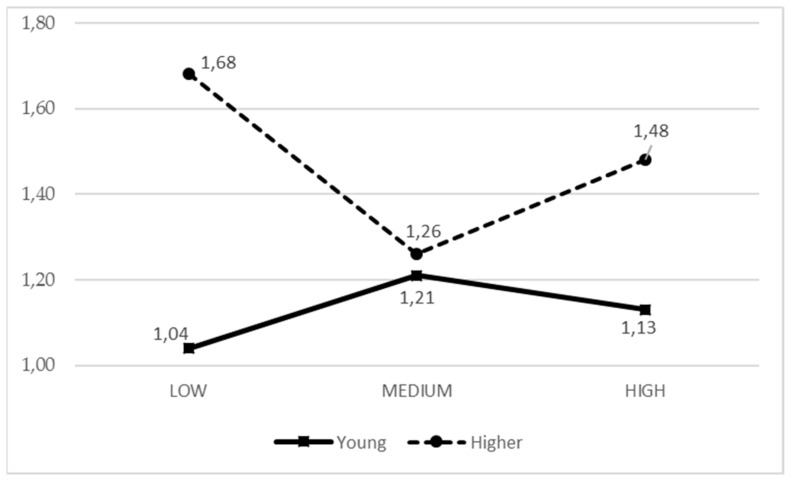
Means of the IPAF4 parental style based on the adolescent age for alcohol consumption.

**Figure 6:**
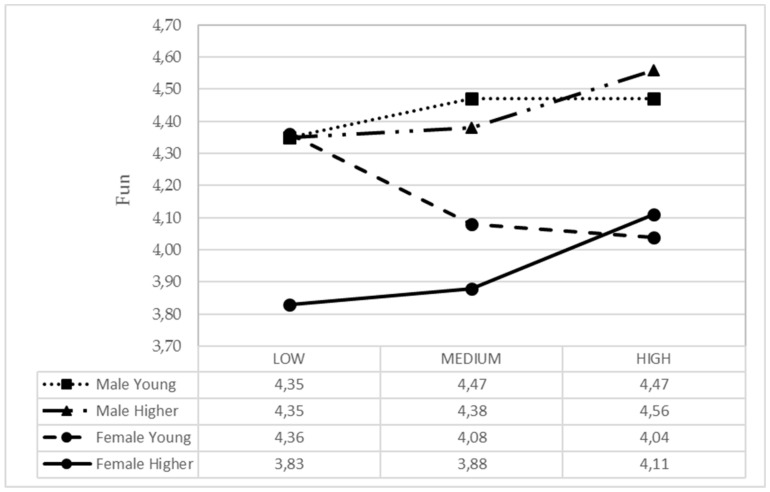
Means of the IPAF4 parental style based on the adolescent gender and age for fun.

**Figure 7:**
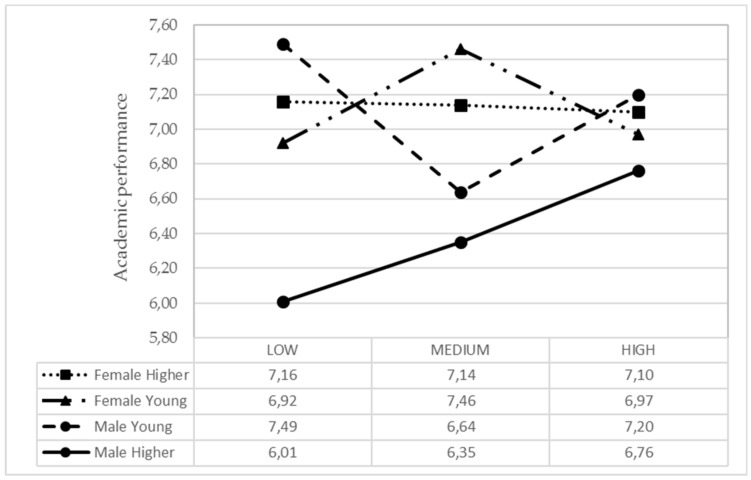
Means of the IPAF4 parental style based on the adolescent gender and age for academic performance.

In younger adolescents, alcohol consumption is lower and independent from parental guidance support. However, those of older age, it was observed that those with lower parental support drink more alcohol than those with medium and higher parental support (Figure 5). There were clear differences between boys and girls concerning fun, depending on whether they were younger or older, depending on IPAF4. In the group of young people, if they had greater parental support, they experienced more fun in the case of boys and less in the case of girls (Figure 6). In the young group, girls showed more academic performance when they received average parental support. On the contrary, young boys got better academic performance when parental support was low or high. In the older group, girls had more academic performance than boys, and regardless of parental support. In the case of older boys, the higher parental support, the better the academic performance (Figure 7).

## 4. Discussion

### 4.1. Correlation between Parental Support Factors, Age and Gender with the Variables of Fun and Foredom

Although the adolescents who have more fun in this study were those who felt more supported by their parents, parental support did not relate equally the feeling of fun or boredom depending on gender and age. Thus, within the group of young people, high levels of parental support in boys equaled more fun, but the same did not happen in the case of girls. These results partially coincided with other researches, where adolescents who appreciated more support from their parents felt more motivated towards PA practice, feeling with it greater enjoyment and fun [49,50,51], in line with the results of this study where the lower parental support towards PA increased the feeling of boredom of children. Consequently, it is convenient for parents to support their children in PA practice as a fun activity and not as a search for performance [52], since it is proven that fun physical-sports practice among young people positively influences future PA practice [6]. Several studies show that parents who support and get involved in PA practice manage to increase their motivation and reduce their children’s boredom, thus favoring adherence to PA [53].

Additionally, the support with material and economic resources was correlated to greater motivation and enjoyment since the adolescents felt more satisfied with PA practice carried out [49]. It was also verified that the subjective well-being of having fun during PA practice generates greater adherence [54], considering the desire to have fun as one of the main and most important reasons why adolescents perform physical activity [55,56].

### 4.2. Correlation between Parental Support Factors, Age and Gender with the Variable of Physical Activity

According to this study, adolescents who have little general parental support are less physically active, which coincides with previous findings where teenagers who felt more support from their parents increased their motivation towards PA practice [49,50,51]. In addition to the importance of the parental support received, which can be logistic, economic or motivational [15], the fact of having physically active parents may influence the PA of their teenage children [22]. In fact, it is proven that a high level of parents’ PA was associated with higher levels of PA practice in their children, up to the age of 24 [57], especially when both parents are active [58]. Even beyond PA practice by parents, there is a very positive significant influence of the parental support and child support self-perceptions, on the adolescent PA adherence [59], which positively results in a good physical condition [60] and even benefits children’s self-esteem [61].

However, parental support affects PA practice in a different way according to the age and gender of adolescents. Thus, in this study, parental support is more related in young boys and more in older girls. In part, this may be explained by the different educational models that children have received from their parents. Therefore, fathers tend to spend more time with their children in sedentary activities and with screen devices, while mothers choose outdoor activities, which seems to have a greater and better influence on healthy habits [17]. Adolescents whose parents are more permissive and democratic feel more motivated towards PE and feel more parental support to practice PA in their free time [62].

### 4.3. Correlation between Parental Support Factors, Age and Gender with the Variable of Academic Performance

Academic performance is related in a different way by parental support according to age and gender. Thus, girls and younger adolescents have better academic performance. More specifically, among younger girls, show more performance when they have medium parental support while boys obtained them when the support is high. There is no clear agreement as to the influence that parental support has on academic performance. There are researches stating that if parents are very supportive of their children expecting academic successes from them and that they build a future for themselves, this may in some cases negatively impact their academic performance [63]. On the other hand, there are investigations considering that in addition to parental support, parental education seems to be a very important variable, since the children of those parents with a higher level of education obtain better grades [64]. In fact, when children receive parental support through an understanding educational style, they obtain better academic performance than if their parents are authoritarian [65].

Furthermore, PA practice correlates positively with academic performance, so it is advisable to reduce the time allocated to the use of screens, in order to maximize the positive effects that healthy habits have on academic performance [1]. Especially because parental support towards PA practice acquires greater importance since it has been confirmed that a higher energy expenditure or level of PA practice correlates positively with the academic performance of schoolchildren [24,66,67].

Consequently, it seems necessary to take into account the influence exerted by the motivation and PA practice on school grades. For this purpose, a positive association has been proved for both gender between the attraction to PA and the academic performance obtained [68]. For instance, according to the data from this study, girls have better academic performance than boys, which may be explained by the greater attention given to the academic issues they show compared to the boys [69,70].

Therefore, it is necessary to reinforce parental support and encourage the collaboration of school and family if the academic performance of children is to be improved [71], because it has been proven that regardless of the gender, age and ethnic group of schoolchildren, parental involvement is considered to be positive [72]. In fact, high parental support is associated with greater school satisfaction and better academic performance, also influencing the involvement of parents in the school adjustment of their children [73].

### 4.4. Correlation between Parental Support Factors, Age and Gender with the Variable of Alcohol Consumption

Although some investigations indicated that both the adolescent’s individual PA practice, as well as the practice accompanied by their parents, did not seem to have a very clear relationship with alcohol consumption [74], it has recently been proven that the parental lifestyle and influence can be decisive in children’s alcohol consumption [75]. In fact, it is known that when parents have healthy habits or do not support their children’s alcohol intake, they have a lower consumption [76]. In addition, alcohol consumption is reduced to a greater extent among children of parents who have a restrictive attitude towards the access to alcohol [77]. Therefore, parents have a fundamental role in preventing and reducing their children’s alcohol consumption [38,76,78]. There are even researches concluding that the perceptions by the children concerning their parents’ alcohol intake, positively predispose them to alcohol consumption [74].

At the same time, parental support and alcohol consumption varies depending on gender. If girls have a bad relationship or influence from parents they are predisposed to excessive alcohol consumption. In turn, if boys are physically active, have a good relationship with their parents and the latter have a higher academic education, these factors act as protective variables against excessive alcohol consumption [79]. Hence, the maternal support received is more important in girls [75].

On top of that, alcohol consumption of schoolchildren in this study increased with age, aggravated by the fact that low parental support was associated with increased alcohol intake, which is consistent with previous researches [76]. Accordingly, while bearing in mind the importance of parents in reducing and preventing alcohol consumption [38,78], we must be aware that in adolescence, in addition to family relationships, the peer group exercises a clear influence on alcohol consumption among young people [80]. Actually, among young people alcohol consumption is not socially penalized and abstemious people are not seen as positive examples [81].

### 4.5. Final Synthesis

Currently, adolescents spend many hours using electronic devices, so at an earlier age the example and influence of physically active parents is greater and has more effect [16], especially if we want PA to be perceived positively and generate adherence [82,83,84], and even more taking into account the important influence that the peer group has on PA practice by adolescents [85]. Consequently, it is important to make parents see that the support they provide to their children is essential to generate adherence to PA practice, being aware that such influence may appear in different ways [58]. Some of them indicate that parents act as a model and children replicate their behaviors while being physically active [14]. Others are related to the parents developing a stimulating function towards PA practice [14,15] or being the economic or logistical support that encourages and promotes the adoption of healthy habits [15]. Without forgetting that parenting style may influence the well-being and self-esteem of their children. For example, greater personal well-being of children is associated with greater parental warmth, that is, a less rigid and authoritative education [86].

## 5. Conclusions

Parental support, whether as a stimulus, model or support, is related to the adoption of healthy habits, enjoyment with physical-sports practice and improves academic performance. Likewise, gender and age are related to the parental support perceived with physical-sports enjoyment, the level of PA practice, the academic performance and the alcohol consumption. Additionally, taking into consideration the current increase in the use of screens by adolescents, it is vital to design joint work programs between schools and families, in order to promote healthy habits, improve academic performance and achieve social and psychological benefits in schoolchildren. In a nutshell, it is transcendental that parents become aware of the importance of parental support, especially at an early age. This support has a positive impact on fields such as healthy habits, academic performance and satisfaction with physical and sports practice.

Due to its design, this study did not permit the extraction of causality relationships between the analyzed variables.

For future research, it is suggested that socioeconomic status should be included as a confounding variable between parental support and the rest of the variables studied.

## Figures and Tables

**Table 1:** Descriptive analysis of the dependent variables of boredom, fun, physical activity (PA) practice, academic performance and alcohol consumption. Analysis according to the factors of gender, age and Parental Support for Physical Activity Questionnaire (APAF).

Factor	Classification		1 Boredom	2 Fun	3 Physical Activity	4 Academic Performance	5 Alcohol Consumption	*p* (Significance)
**SEX**	Male (*n* = 494)	M	1.99	4.44	3.62	6.78	1.30	(1) *p* < 0.001 (2) *p* < 0.001 (3) *p* < 0.001 (4) *p* < 0.001
		SD	0.76	0.64	1.78	1.42	0.73	
	Female (*n* = 606)	M	2.24	4.05	2.83	7.07	1.32	
		SD	0.81	0.76	1.68	1.46	0.730	
AGE	Young (*n* = 600)	M	2.06	4.28	3.39	7.10	1.12	(1) *p* = 0.003 (2) *p* < 0.001 (3) *p* < 0.001 (4) *p* < 0.001 (5) *p* < 0.001
		SD	0.87	0.73	1.78	1.34	0.47	
	Higher (*n* = 500)	M	2.20	4.16	2.94	6.76	1.54	
		SD	0.69	0.74	1.72	1.56	0.90	
APAF1	Low (*n* = 294)	M	2.27	3.91	2.56	6.80	1.39	(1) *p* < 0.001 (2) *p* < 0.001 (3) *p* < 0.001 (4) *p* = 0.031 (5) *p* < 0.001
		SD	0.80	0.86	1.91	1.55	0.80	
	Medium (*n* = 520)	M	2.15	4.22	3.29	6.90	1.30	
		SD	0.78	0.67	1.72	1.39	0.71	
	High (*n* = 286)	M	1.92	4.56	3.62	7.17	1.25	
		SD	0.80	0.52	1.54	1.43	0.69	
APAF2	Low (*n* = 382)	M	2.13	4.13	2.95	6.96	1.43	(2) *p* = 0.006 (5) *p* < 0.001
		SD	0.74	0.82	1.73	1.43	0.84	
	Medium (*n* = 278)	M	2.05	4.23	3.28	6.97	1.22	
		SD	0.68	0.67	1.88	1.53	0.61	
	High (*n* = 400)	M	2.17	4.31	3.32	6.91	1.26	
		SD	0.91	0.68	1.72	1.42	0.68	
APAF3	Low (*n* = 348)	M	2.14	4.16	3.05	7.02	1.40	(1) *p* = 0.003 (4) *p* = 0.003 (5) *p* = 0.002
		SD	0.77	0.81	1.85	1.49	0.817	
	Medium (*n* = 242)	M	2.00	4.25	3.26	7.12	1.36	
		SD	0.80	0.73	1.61	1.45	0.771	
	High (*n* = 510)	M	2.18	4.26	3.24	6.80	1.23	
		SD	0.81	0.68	1.79	1.41	0.629	
APAF4	Low (*n* = 428)	M	2.08	4.18	2.98	6.87	1.41	(1) *p* = 0.001
		SD	0.76	0.78	1.89	1.55	0.82	
	Medium (*n* = 178)	M	2.00	4.19	3.38	6.94	1.24	
		SD	0.64	0.71	1.61	1.52	0.66	
	High (*n* = 494)	M	2.21	4.28	3.29	7.01	1.25	
		SD	0.87	0.70	1.70	1.33	0.66	

APAF1: general parental support; APAF2: active parents; APAF3: past parental activity; APAF4: guidance support. M: average; SD: standard deviation, PA: physical activity; RA: academic performance; *p*: significance. Note: in the column of *p* (sig) the number in brackets “()” corresponds to the independent variables (numbered 1–5). Note: the M and SD value is weighted according to the response scale corresponding to each of the independent variables analyzed: Boredom and fun (from the SSI questionnaire) with Likert-type response scale ranging from 1 (“strongly disagree”) to 5 (“strongly agree”); PA from 1 to 7 days; RA of a score from 0 to 10; Alcohol consumption with a response scale ranging from 1 does not consume, 2 low consumption, 3 average consumption and 4 excessive consumption. Young (12, 13 and 14 years) and higher (15 and 16 years).

**Table 2:** Multivariate analysis of APAF factors, gender and age and the dependent variables of boredom, fun, PA practice, academic performance and alcohol consumption.

Factor		Boredom		Fun		Physical Activity		Academic Performance		Alcohol Consumption	
	ʌ	*p*	η^2^	*p*	η^2^	*p*	η^2^	*p*	η^2^	*p*	η^2^
(B) Gender	0.918	<0.001	0.016	<0.001	0.041	<0.001	0.038	<0.001	0.014	0.605	<0.001
(C) Age	0.896	0.003	0.008	<0.001	0.003	<0.001	0.013	<0.001	0.013	<0.001	0.082
B × C	0.955	0.254	0.001	0.002	0.008	0.290	0.001	<0.001	0.026	0.179	0.002
(A1) APAF1	0.889	<0.001	0.015	<0.001	0.090	<0.001	0.034	0.031	0.006	0.953	<0.001
A1 × B	0.963	<0.001	0.017	0.013	0.008	0.041	0.006	0.115	0.004	0.072	0.005
A1 × C	0.976	0.018	0.007	0.064	0.005	0.471	0.001	0.060	0.005	0.247	0.003
A1 × B × C	0.964	0.359	0.002	<0.001	0.019	0.926	<0.001	0.042	0.006	0.041	0.006
(A2) APAF2	0.963	0.172	0.003	0.006	0.009	0.089	0.004	0.432	0.002	<0.001	0.013
A2 × B	0.911	0.933	<0.001	0.684	0.001	0.904	<0.001	0.749	0.001	0.020	0.007
A2 × C	0.963	0.001	0.012	0.019	0.007	0.059	0.005	0.935	<0.001	<0.001	0.018
A2 × B × C	0.983	0.528	0.001	0.094	0.004	0.178	0.003	0.270	0.002	0.077	0.005
(A3) APAF3	0.964	0.003	0.011	0.261	0.002	0.282	0.002	0.003	0.011	0.002	0.011
A3 × B	0.900	0.128	0.004	0.842	<0.001	0.862	<0.001	0.163	0.003	0.376	0.002
A3 × C	0.961	0.006	0.009	0.467	0.001	0.972	<0.001	0.001	0.013	<0.001	0.018
A3 × B × C	0.976	0.041	0.006	0.025	0.007	0.001	0.013	0.182	0.003	0.287	0.002
(A4) APAF4	0.962	0.001	0.012	0.200	0.003	0.127	0.004	0.491	0.001	0.109	0.004
A4 × B	0.979	0.433	0.002	0.118	0.004	0.233	0.003	0.015	0.008	0.619	0.001
A4 × C	0.947	<0.001	0.029	0.019	0.007	0.813	<0.001	0.660	0.005	<0.001	0.023
A4 × B × C	0.966	0.499	0.001	0.001	0.012	0.654	0.001	0.001	0.013	0.068	0.005

ʌ: Wilks’ lambda for multivariate analysis; *p*: significance; η^2^: Partial eta squared. APAF1: general parental support; APAF2: active parents; APAF3: past parental activity; APAF4: guidance support.

## References

[1] Aguilar M.M., Vergara F.A., Velásquez E.J.A., Marina R., García-Hermoso A. (2015). Screen time impairs the relationship between physical fitness and academic attainment in children. J. Pediatr. (Rio. J.).

[2] Mitchell J., Skouteris H., McCabe M., Ricciardelli L.A., Milgrom J., Baur L.A., Fuller-Tyszkiewicz M., Dwyer G. (2012). Physical activity in young children: A systematic review of parental influences. Early Child Dev. Care.

[3] Granero-Gallegos A., Baena-Extremera A., Sánchez-Fuentes J.A., Martínez-Molina M. (2014). Motivational profiles of autonomy support, self-determination, satisfaction, importance of physical education and intention to partake in leisure time physical activity. Cuad. Psicol. del Deport..

[4] Baños R., Barretos-Ruvalcaba M., Baena-Extremera A. (2019). Protocol to study the academic, psychological and physical activity variables that influence the academic performance of Mexican and Spanish adolescents. Espiral. Cuad. del Profr..

[5] Ruiz-Juan F., Baños R., Fuentesal-Garcia J., García-Montes E., Baena-Extremera A. (2019). Transcultural analysis of motivational climate in students in Costa Rica, Mexico and Spain. Rev. Int. Med. y Ciencias la Act. Fis. y del Deport..

[6] Baños R., Marentes M., Zamarripa J., Baena-Extremera A., Ortiz-Camacho M., Duarte-Félix H. (2019). Influence of satisfaction, boredom and importance of extracurricular physical education in Mexican adolescents. Cuad. Psicol. del Deport..

[7] Candela F., Zucchetti G., Villosio C. (2014). Preliminary validation of the Italian version of the original sport motivation scale. J. Hum. Sport Exerc..

[8] Bandura A. (1998). Health promotion from the perspective of social cognitive theory. Psychol. Heal..

[9] Deci E.L., Ryan R.M. (1990). A motivational approach to self: Integration in personality. Nebr. Symp. Motiv..

[10] Moral-Garcia J.E., Román-Palmero J., García S.L., Guillamón A.R., Soto J.J.P., Cantó E.G. (2019). Psychometric properties of the Sports Motivation Scale, and analysis of motivation in physical education classes and its relationship with out-of-school physical activity levels. Retos.

[11] Ruiz-Ariza A., De La Torre-Cruz M.J., Suárez-Manzano S., Martínez-López E.J. (2019). Support towards physical activity and academic performance regardless of parental socio-educational status. Retos.

[12] Bauer K.W., Nelson M.C., Boutelle K.N., Neumark-Sztainer D. (2008). Parental influences on adolescents’ physical activity and sedentary behavior: Longitudinal fndings from Project EAT-II. Int. J. Behav. Nutr. Phys. Act..

[13] Kwon S., Janz K.F., Letuchy E.M., Burns T.L., Levy S.M. (2016). Parental characteristic patterns associated with maintaining healthy physical activity behavior during childhood and adolescence. Int. J. Behav. Nutr. Phys. Act..

[14] Olivares P.R., Cossio-Bolaños M.A., Gomez-Campos R., Almonacid-Fierro A., Garcia-Rubio J. (2015). Influence of parents and physical education teachers in adolescent physical activity. Int. J. Clin. Heal. Psychol..

[15] Mendonça G., Cheng L.A., Mélo E.N., De Farias Júnior J.C. (2014). Physical activity and social support in adolescents: A systematic review. Health Educ. Res..

[16] Brzęk A., Strauss M., Przybylek B., Dworrak T., Dworrak B., Leischik R. (2018). How does the activity level of the parents influence their children’s activity? The contemporary life in a world ruled by electronic devices. Arch. Med. Sci..

[17] Schoeppe S., Vandelanotte C., Bere E., Lien N., Verloigne M., Kovacs E., Van Lippevelde W. (2016). The influence of parental modelling on children’s physical activity and screen time: Does it differ by gender?. Eur. J. Public Health.

[18] Jacobi D., Caille A., Borys J.M., Lommez A., Couet C., Charles M.A., Oppert J.M., FLVS Study Group (2011). Parent-offspring correlations in pedometerassessed physical activity. PLoS ONE.

[19] Rosa Guillamon A., Garcia Canto E., Rodríguez García P.L., Pérez Soto J.J., Tárraga Marcos M.L., Tárraga López P.J. (2017). Physical activity, physical fitness and quality of diet in schoolchildren from 8 to 12 years. Nutr. Hosp..

[20] Ortega F.B., Ruiz J.R., Castillo M.J. (2013). Physical activity, physical fitness, and overweight in children and adolescents: Evidence from epidemiologic studies. Endocrinol. y Nutr..

[21] Guillamón A.R., Canto E.G., López P.J.C. (2019). Physical activity, physical fitness and self-concept in schoolchildren aged between 8 to 12 years old. Retos.

[22] Sánchez-Zamorano L.M., Solano-González M., Macias-Morales N., Flores-Sánchez G., Galván-Portillo M.V., Lazcano-Ponce E.C. (2019). Perception of parents’ physical activity as a positive model on physical activity of adolescents. Prev. Med. (Baltim)..

[23] Scudder M.R., Federmeier K.D., Raine L.B., Direito A., Boyd J.K., Hillman C.H. (2014). The association between aerobic fitness and language processing in children: Implications for academic achievement. Brain Cogn..

[24] Álvarez-Bueno C., Pesce C., Cavero-Redondo I., Sánchez-López M., Martínez-Hortelano J.A., Martínez-Vizcaíno V. (2017). The Effect of Physical Activity Interventions on Children’s Cognition and Metacognition: A Systematic Review and Meta-Analysis. J. Am. Acad. Child Adolesc. Psychiatry.

[25] Chaddock-Heyman L., Erickson K.I., Voss M.W., Knecht A.M., Pontifex M.B., Castelli D.M., Hillman C.H., Kramer A.F. (2013). The effects of physical activity on functional MRI activation associated with cognitive control in children: A randomized controlled intervention. Front. Hum. Neurosci..

[26] De los Mozos-Huertas J.L. (2018). Physical condition and academic performance. J. Sport Heal. Res..

[27] Ramos P., Jiménez-Iglesias A., Rivera F., Moreno C. (2016). Physical activity trends in spanish adolescents. Rev. Int. Med. y Ciencias la Act. Fis. y del Deport..

[28] Dale H., Brassington L., King K. (2014). The impact of healthy lifestyle interventions on mental health and wellbeing: A systematic review. Ment. Heal. Rev. J..

[29] Hoare E., Milton K., Foster C., Allender S. (2016). The associations between sedentary behaviour and mental health among adolescents: A systematic review. Int. J. Behav. Nutr. Phys. Act..

[30] Telama R., Yang X., Leskinen E., Kankaanpää A., Hirvensalo M., Tammelin T., Viikari J.S.A., Raitakari O.T. (2014). Tracking of physical activity from early childhood through youth into adulthood. Med. Sci. Sports Exerc..

[31] Patton G.C., Sawyer S.M., Santelli J.S., Ross D.A., Afifi R., Allen N.B., Arora M., Azzopardi P., Baldwin W., Bonell C. (2016). Our future: A Lancet commission on adolescent health and wellbeing. Lancet.

[32] Hall W.D., Patton G., Stockings E., Weier M., Lynskey M., Morley K.I., Degenhardt L. (2016). Why young people’s substance use matters for global health. Lancet Psychiatry.

[33] Degenhardt L., Stockings E., Patton G., Hall W.D., Lynskey M. (2016). The increasing global health priority of substance use in young people. Lancet Psychiatry.

[34] Mattick R.P., Clare P.J., Aiken A., Wadolowski M., Hutchinson D., Najman J., Slade T., Bruno R., McBride N., Kypri K. (2018). Association of parental supply of alcohol with adolescent drinking, alcohol-related harms, and alcohol use disorder symptoms: A prospective cohort study. Lancet Public Health.

[35] Homel J., Warren D. (2019). The Relationship between Parent Drinking and Adolescent Drinking: Differences for Mothers and Fathers and Boys and Girls. Subst. Use Misuse.

[36] Smorti M., Guarnieri S. (2015). The parental bond and alcohol use among adolescents: The mediating role of drinking motives. Subst. Use Misuse.

[37] Moral García J.E., Agraso López A.D., Pérez Soto J.J., Rosa Guillamón A., Tárraga Marcos M.L., García Cantó E., Tárraga López P.J. (2019). Phisical activity practice according to adherence to the Mediterranean diet, alcohol consumption and motivation in adolescents. Nutr. Hosp..

[38] Yap M.B.H., Cheong T.W.K., Zaravinos-Tsakos F., Lubman D.I., Jorm A.F. (2017). Modifiable parenting factors associated with adolescent alcohol misuse: A systematic review and meta-analysis of longitudinal studies. Addiction.

[39] Jago R., Fox K.R., Page A.S., Brockman R., Thompson J.L. (2009). Development of scales to assess children’s perceptions of friend and parental influences on physical activity. Int. J. Behav. Nutr. Phys. Act..

[40] Brockman R., Jago R., Fox K.R., Thompson J.L., Cartwright K., Page A.S. (2009). “Get off the sofa and go and play”: Family and socioeconomic influences on the physical activity of 10–11 year old children. BMC Public Health.

[41] Jago R., Brockman R., Fox K.R., Cartwright K., Page A.S., Thompson J.L. (2009). Friendship groups and physical activity: Qualitative findings on how physical activity is initiated and maintained among 10–11 year old children. Int. J. Behav. Nutr. Phys. Act..

[42] Baena-Extremera A., Granero-Gallegos A., Bracho-Amador C., Pérez-Quero F.J. (2012). Spanish version of the sport satisfaction instrument (SSI) adapted to physical education. Rev. Psicodidact..

[43] Prochaska J.J., Sallis J.F., Long B. (2001). A physical activity screening measure for use with adolescents in primary care. Arch. Pediatr. Adolesc. Med..

[44] Martínez-López E.J., Hita-Contreras F., Moral-García J.E., Grao-Cruces A., Ruiz J.R., Redecillas-Peiró M.T., Martínez-Amat A. (2015). Association of low weekly physical activity and sedentary lifestyle with self-perceived health, pain, and well-being in a Spanish teenage population. Sci. Sport..

[45] Martinez-López E.J., Moreno-Cerceda J., Suarez-Manzano S., Ruiz-Ariza A. (2018). Effect of and satisfaction with a program of physical activity controlled through heart rate monitors on body mass index in young students with overweight-obesity. Retos.

[46] Saunders J.B., Aasland O.G., Babor T.F., de la Fuente J.R., Grant M. (1993). Development of the Alcohol Use Disorders Identification Test (AUDIT): WHO Collaborative Project on Early Detection of Persons with Harmful Alcohol Consumption–II. Addiction.

[47] Rubio Valladoli G., Bermejo Vicedo J., Caballero Sanchez-Serrano M.C., Santo-Domingo C.J. (1998). Validation of the test for the identification of alcohol use disorders (AUDIT) in primary care. Rev. Clínica Española.

[48] Ley Orgánica 15/1999, de 13 de diciembre, de Protección de Datos de Carácter Personal. https://www.boe.es/buscar/doc.php?id=BOE-A-1999-23750.

[49] De la Torre-Cruz M.J., Ruiz-Ariza A., Suárez-Manzano S., Martínez-López E.J. (2019). Perceived parental support and adolescents’ motivation toward physical activity. Rev. Psicol. del Deport..

[50] Sebire S.J., Haase A.M., Montgomery A.A., McNeill J., Jago R. (2014). Associations between physical activity parenting practices and adolescent girls’ self-perceptions and physical activity intentions. J. Phys. Act. Health.

[51] Verloigne M., Veitch J., Carver A., Salmon J., Cardon G., De Bourdeaudhuij I., Timperio A. (2014). Exploring associations between parental and peer variables, personal variables and physical activity among adolescents: A mediation analysis. BMC Public Health.

[52] Logan K., Cuff S., LaBella C.R., Brooks M.A., Canty G., Diamond A.B., Hennrikus W., Moffatt K., Nemeth B.A., Pengel K.B. (2019). Organized sports for children, preadolescents, and adolescents. Pediatrics.

[53] Sánchez-Miguel P.A., González J.J.P., Alonso D.A., Marcos F.M.L., Sánchez-Oliva D., Ponce I.G. (2015). Profiles of parental behavior in sport and its relationship with their children’ motivational processes. Motricidade.

[54] Videra-García A., Reigal-Garrido R. (2013). Physical self-concept, perceptions of health and life satisfaction in a sample of adolescents. An. Psicol..

[55] Granero-Gallegos A., Baena-Extremera A., Pérez-Quero F.J., Ortiz-Camacho M.M., Bracho-Amador C. (2012). Analysis of motivational profiles of satisfaction and importance of physical education in high school adolescents. J. Sport. Sci. Med..

[56] Baena-Extremera A., Granero-Gallegos A., Ponce-de-León-Elizondo A., Sanz-Arazuri E., Valdemoros-San-Emeterio M.Á., Martínez-Molina M. (2016). Psychological factors related to physical education classes as predictors of students’ intention to partake in leisure-time physical activity. Cienc. e Saude Coletiva.

[57] Kaseva K., Hintsa T., Lipsanen J., Pulkki-Raback L., Hintsanen M., Yang X., Hirvensalo M., Hutri-Kähönen N., Raitakari O., Keltikangas-Järvinen L. (2017). Parental physical activity associates with offspring’s physical activity until middle age: A 30-year study. J. Phys. Act. Heal..

[58] Marques A., Valeiro M.G., Martins J., Fernndez-Villarino M.A., Da Costa F.C. (2017). Relationship between physical activity of adolescents and that of mothers/parents. Rev. Psicol. del Deport..

[59] Wilk P., Clark A.F., Maltby A., Tucker P., Gilliland J.A. (2018). Exploring the effect of parental influence on children’s physical activity: The mediating role of children’s perceptions of parental support. Prev. Med..

[60] Carbert N.S., Brussoni M., Geller J., Mâsse L.C. (2019). Familial Environment and Overweight/Obese Adolescents’ Physical Activity. Int. J. Environ. Res. Public Health.

[61] Qurban H., Wang J., Siddique H., Morris T., Qiao Z. (2019). The mediating role of parental support: The relation between sports participation, self-esteem, and motivation for sports among chinese students. Curr. Psychol..

[62] Martínez-López E.J., López-Leiva F., Moral-García J.E., De la Torre-Cruz M.J. (2014). Parental styles and indicators of physical activity in children and adolescents. Behav. Psychol. Psicol. Conduct..

[63] Hill N.E., Liang B., Bravo D.Y., Price M., Polk W., Perella J., Savitz-Romer M. (2018). Adolescents’ Perceptions of the Economy: Its Association with Academic Engagement and the Role of School-Based and Parental Relationships. J. Youth Adolesc..

[64] Crede J., Wirthwein L., McElvany N., Steinmayr R. (2015). Adolescents’ academic achievement and life satisfaction: The role of parents’ education. Front. Psychol..

[65] García F., Serra E., García O.F., Martínez I., Cruise E. (2019). A Third Emerging Stage for the Current Digital Society? Optimal Parenting Styles in Spain, the United States, Germany, and Brazil. Int. J. Environ. Res. Public Health.

[66] Reloba-Martínez S., Reigal-Garrido R.E., Hernández-Mendo A., Martínez-López E.J., Martín-Tamayo I., Chirosa-Ríos L.J. (2017). Effects of after-school, high-intensity physical activity programme, on levels of attention of school children. Rev. Psicol. del Deport..

[67] Wang P.-S., Huang Y.-C., Wu S.-F.V., Wang K.-M. (2014). Effects of daily energy expenditure on academic performance of elementary students in Taiwan. Japan J. Nurs. Sci..

[68] Ruiz-Ariza A., Ruiz J.R., De La Torre-Cruz M., Latorre-Román P., Martínez-López E.J. (2016). Influence of level of attraction to physical activity on academic performance of adolescents. Rev. Latinoam. Psicol..

[69] Hernando A., Oliva A., Pertegal M.-A. (2012). Family variables and academic achievement in adolescence. Estud. Psicol..

[70] Berge J.M., Wall M., Larson N., Loth K.A., Neumark-Sztainer D. (2013). Family functioning: Associations with weight status, eating behaviors, and physical activity in adolescents. J. Adolesc. Heal..

[71] Hill N.E., Sheridan S., Moorman K.E. (2015). Family school relationships duringadolescence clarifying goals broadening conceptualizations and deepening impact. Processes and Pathways of Family-School Partnerships Across Development. Research on Family-School Partnerships.

[72] Wilder S. (2014). Effects of parental involvement on academic achievement: A meta-synthesis. Educ. Rev..

[73] Serna C., Martínez I. (2019). Parental Involvement as a Protective Factor in School Adjustment among Retained and Promoted Secondary Students. Sustainability.

[74] Pentz M.A., Riggs N.R. (2013). Longitudinal Relationships of Executive Cognitive Function and Parent Influence to Child Substance Use and Physical Activity. Prev. Sci..

[75] De Albéniz-Garrote G.P., Rubio-Rubio L., Medina-Gómez B. (2018). The moderating role of parenting styles in the relationship between impulsivity and alcohol consumption in a sample of Spanish adolescents. Rev. Psicopatol. y Psicol. Clin..

[76] Shaw T., Johnston R.S., Gilligan C., McBride N., Thomas L.T. (2018). Child-parent agreement on alcohol-related parenting: Opportunities for prevention of alcohol-related harm. Heal. Promot. J. Aust..

[77] Tael-Öeren M., Naughton F., Sutton S. (2019). The relationship between parental attitudes and children’s alcohol use: A systematic review and meta-analysis. Addiction.

[78] Kaynak Ö., Winters K.C., Cacciola J., Kirby K.C., Arria A.M. (2014). Providing alcohol for underage youth: What messages should we be sending parents?. J. Stud. Alcohol Drugs.

[79] Cerkez I., Culjak Z., Zenic N., Sekulic D., Kondric M. (2015). Harmful Alcohol Drinking Among Adolescents: The Influence of Sport Participation, Religiosity, and Parental Factors. J. Child Adolesc. Subst. Abus..

[80] Lo Y., Chen W.-T., Wang I.-A., Liu C.-Y., Chen W.J., Chen C.-Y. (2019). Family and school social capitals in late childhood predict youthful drinking behaviors and problems. Drug Alcohol Depend..

[81] Jones S.C., Andrews K., Berry N. (2016). Lost in translation: A focus group study of parents’ and adolescents’ interpretations of underage drinking and parental supply. BMC Public Health.

[82] Pelegrín A., García H.G., De Los Fayos E.J.G. (2019). Perception of parents’ education style in adolescents, physical activity practitioners, federation members, and players. Retos.

[83] González-García H., Pelegrín A., Carballo J.L. (2018). Parental educational styles as a predictor of sport success and sports competition level. Rev. Int. Med. y Ciencias la Act. Fis. y del Deport..

[84] Yao C.A., Rhodes R.E. (2015). Parental correlates in child and adolescent physical activity: A meta-analysis. Int. J. Behav. Nutr. Phys. Act..

[85] Haidar A., Ranjit N., Archer N., Hoelscher D.M. (2019). Parental and peer social support is associated with healthier physical activity behaviors in adolescents: A cross-sectional analysis of Texas School Physical Activity and Nutrition (TX SPAN) data. BMC Public Health.

[86] Garcia O.F., Serra E. (2019). Raising Children with Poor School Performance: Parenting Styles and Short- and Long-Term Consequences for Adolescent and Adult Development. Int. J. Environ. Res. Public Health.

